# Longevity and Magnitude of Antibody Responses After Homologous and Heterologous COVID-19 Booster Vaccinations in Bangladesh

**DOI:** 10.3390/vaccines14060531

**Published:** 2026-06-15

**Authors:** Marjahan Akhtar, Md. Rashedul Islam, Zahid Hasan Khan, Afroza Akter, Imam Tauheed, Tasnuva Ahmed, Ishtiakul Islam Khan, Mohammad Ashraful Amin, Fatema Khaton, Farhana Khanam, Md. Taufiqul Islam, Prasanta Kumar Biswas, Rumana Rashid, Md. Mamunur Rashid, Md. Zakir Hossain, Ahmed Nawsher Alam, A. S. M. Alamgir, Edward T. Ryan, Sayera Banu, Tahmina Shirin, Fahima Chowdhury, Ashraful Islam Khan, Taufiqur Rahman Bhuiyan, Firdausi Qadri

**Affiliations:** 1 Infectious Diseases Division, International Centre for Diarrhoeal Disease Research Bangladesh (icddr,b), Dhaka 1212, Bangladeshtaufiqur@icddrb.org (T.R.B.); fqadri@icddrb.org (F.Q.); 2 Bangladesh Institute of Tropical & Infectious Diseases (BITID), Chittagong 4316, Bangladesh; 3 Institute of Epidemiology, Disease Control and Research (IEDCR), Dhaka 1212, Bangladesh; 4 Division of Infectious Diseases, Massachusetts General Hospital, Boston, MA 02214, USA; 5 Department of Medicine, Harvard Medical School, Boston, MA 02215, USA; 6 Department of Immunology and Infectious Diseases, Harvard T.H. Chan School of Public Health, Boston, MA 02215, USA

**Keywords:** COVID-19, vaccination, booster dose, heterologous boosting, antibody response, IgG kinetics

## Abstract

Background: The dynamics of humoral immune responses following primary and booster COVID-19 vaccinations are crucial to understand in order to optimize vaccination strategies. This study evaluates the magnitude and durability of SARS-CoV-2-specific IgG antibody responses across different vaccines in a large cohort of Bangladeshi adults. Methods: A total of 6300 adults from nine hospitals across eight divisions of Bangladesh were enrolled. Participants received two primary doses of either ChAdOx1 nCoV-19 (Covishield, Serum Institute of India, n = 2855), mRNA-1273 (Moderna, n = 578), BNT162b2 (Pfizer-BioNTech, n = 121), or Vero-cell-inactivated (Sinopharm, n = 2746) vaccines. Booster doses were administered at one-year intervals post-primary vaccination. SARS-CoV-2 spike receptor-binding domain (RBD)-specific IgG antibody responses were measured by ELISA using serum from vaccinees at multiple time points after two primary and two booster doses. Results: A total of 3745 individuals received booster 1 (third dose), with 59% receiving heterologous boosters (a different vaccine regimen than the primary doses). Only 5.5% (n = 347) of participants received a second booster one year after the first booster (among them, 99% received BNT162b2). Our results suggest that heterologous boosters with the mRNA vaccine induced higher IgG levels than homologous boosters for individuals who received primary vaccination with adenovirus vector-based ChAdOx1 nCoV-19 or a Vero-cell-inactivated vaccine. However, in those who initially received the mRNA-based vaccine, both homologous and heterologous boosters produced comparable IgG responses. Among all vaccine types, booster immunization with the Vero-cell-inactivated vaccine induced the lowest antibody responses. Longitudinal analysis demonstrated significantly high IgG levels over the 12 months following the first booster (*p* < 0.0001); however, IgG levels declined significantly after the second booster dose (fourth dose). Conclusions: Heterologous boosting strategies, particularly those involving mRNA vaccines, elicit stronger and more sustained IgG responses compared to a homologous booster. However, antibody waning after the second booster highlights the need for continued monitoring and potential additional vaccine strategies.

## 1. Introduction

COVID-19 vaccination is a cornerstone in the global effort to control the SARS-CoV-2 pandemic. In various epidemiological settings, including Bangladesh, individuals who have completed primary COVID-19 vaccination exhibited robust immune responses [1,2]. However, immune responses generated by primary vaccination were not long-lasting, as reduced antibody responses were observed over time [3,4,5]. Despite the administration of two doses of COVID-19 vaccines to the majority of the population, many counties encountered the emergence of novel SARS-CoV-2 variants, especially Delta and Omicron, which demonstrated increased transmissibility and immune evasion capabilities [6,7,8]. Waning immunity along with the resurgence of COVID-19 indicates a need for booster vaccinations to sustain protective immunity, particularly in populations with high disease burdens. In Bangladesh, policymakers decided to focus booster vaccination among the front-line workers and the elderly as priority groups; later, in order to stop the transmission of new variants, administration was broadened to the general population aged 18 years and above. A cross-sectional study among the general Bangladeshi population showed a 70% acceptance rate of the COVID-19 booster [9].

Booster vaccination strategies follow either a homologous approach, administering the same vaccine as the primary doses (e.g., BNT162b2 for both primary and booster), or a heterologous approach, where a different vaccine platform is used from the primary doses (e.g., ChAdOx1 nCoV-19 as the primary and BNT162b2 as the booster vaccine). The most common COVID-19 vaccines used in Bangladesh were ChAdOx1 nCoV-19 (Serum Institute of India), mRNA-1273 (Moderna), BNT162b2 (Pfizer-BioNTech, and Vero-cell-inactivated vaccines (Sinopharm) (https://dashboard.dghs.gov.bd/pages/covid19-vaccination-update.php, accessed on 1 January 2025). Booster vaccination has been shown to enhance both humoral and cellular immune responses, increasing antibody titers, memory B cell persistence, and T-cell functionality, thereby improving long-term protection against pathogens [10]. A homologous booster reinforces immunological memory by stimulating antigen-specific B and T cells. In contrast, a heterologous booster can elicit broader and potentially more durable immune responses due to increased antigenic diversity and cross-stimulation of immune pathways [11,12,13,14]. Studies have shown that heterogenous boosters lead to significantly higher neutralizing antibody levels and enhanced T-cell responses compared to homologous regimens [15,16].

Our group has shown that the administration of ChAdOx1 nCoV-19, mRNA-1273 and BNT162b2 vaccines resulted in a significant increase in spike receptor-binding domain (RBD)-specific antibodies following the initial two doses [1]. The RBD portion of the spike S1 protein has been a particularly attractive immunologic target for COVID-19 vaccine development because of its critical role in binding of the virus to the human angiotensin-converting enzyme 2 (ACE2) receptor, which promotes the entry of SARS-CoV-2 into target cells [17,18,19]. However, the magnitude and duration of these antibody responses are influenced by multiple factors, including the type of vaccine administered, pre-existing immunity from natural infection or prior vaccination, genetic and environmental influences, and underlying nutritional and health conditions [20,21,22,23,24,25]. Given the diverse vaccination landscape in Bangladesh, with populations receiving a mix of inactivated, vector-based, and mRNA vaccines, evaluating the immunogenicity of homologous versus heterologous boosters is crucial. No study has been conducted yet to monitor heterologous and homologous booster vaccine-induced immune responses and their longevity in Bangladesh. This study aims to compare the immune responses induced by homologous and heterologous booster doses in a Bangladeshi cohort, providing critical data to guide evidence-based booster vaccination policies. By elucidating the nature and durability of primary vaccine and booster-induced immunity over 2 years, our findings provide valuable insight into optimizing booster vaccination policies in Bangladesh and similar low- and middle-income country settings.

## 2. Methods and Materials

### 2.1. Study Design

The study used samples collected from 6300 adults enrolled at nine different hospitals located in eight divisions across Bangladesh. These are Kurmitola General Hospital, Mugda Medical College, Mymensingh Medical College, Bangladesh Institute of Tropical and Infectious Diseases, Chattogram, Habiganj District Hospital, Rajshahi Medical College, 250 Bedded General Hospital, Thakurgaon, Satkhira District Hospital, and Patuakhali District Hospital of Bangladesh. Participants who received ChAdOx1 nCoV-19 (Covishield, Serum Institute of India, India), mRNA-1273, BNT162b2 as the primary vaccine, or Vero-cell-inactivated vaccines between March 2021 and November 2021, were enrolled in the study. Participants received two primary doses of the COVID-19 vaccines at an interval of one month (mRNA and Vero-cell-inactivated vaccines) or two months (ChAdOx1 nCoV-19). One year after vaccination, most of the participants received a booster dose. One year after the first booster, a few participants received a second booster. All participants were followed for two years after enrollment. Among the participants, the majority were male (66%, n = 4134). Demographic details of the study participants are shown in Table 1.

The kinetics of immune responses after four primary doses were previously reported (submitted manuscript, 2025). In this analysis, antibody responses were measured after each primary and booster dose. Blood samples were collected from each enrolled participant after each vaccine dose: one/two months after the first dose, one month after the second dose, pre-booster, one month after booster 1, and one month after booster 2. By following the guidelines of the Ministry of Health and Family Welfare, Government of Bangladesh, adult participants received two primary doses of the COVID-19 vaccines at an interval of one/two months. Participants were interviewed to collect demographic and clinical information (e.g., age, sex) prior to enrollment, and data were recorded. Informed written consent was obtained from all participants. The Institutional Review Board (IRB) Committee of the International Centre for Diarrhoeal Disease Research, Bangladesh (icddr,b) and Institute of Epidemiology, Disease Control and Research (IEDCR) approved the study protocol (PR# 21028).

### 2.2. ELISA

Antibody measurement was performed from stored frozen (−20 °C) sera that were separated from blood by differential centrifugation (700× *g* for 15 min). RBD-specific IgG antibodies were determined by ELISA. ELISA plates (96 wells, Nunc^®^ MaxiSorp™, ThermoFisher, Denmark) were coated with 1 μg/mL SARS-CoV-2 RBD antigen (gifts from A. Schmidt lab, Ragon Institute, Boston, MA, USA) and incubated for one hour at room temperature followed by blocking with 5% non-fat milk. Serum samples were heat-inactivated and added to the plates (serially 4-fold diluted samples in 5% Milk- 1X PBS 0.05% Tween). An anti-RBD monoclonal antibody (Mab CR3022, gifts from A. Schmidt lab, Ragon Institute, Boston MA) was added to the plate, 8 serial dilutions were performed, and plates were incubated for an hour at 37 °C. Subsequently, goat anti-human IgG peroxidase-conjugated secondary antibodies (Jackson ImmunoResearch, USA) and ortho phenylenediamine (Sigma, USA) in 0.1 M sodium citrate buffer (pH 4.5) with 30% hydrogen peroxide (Merck, Germany) were added to the plates. Reactions were allowed for 20 min and optical density (OD) was measured at dual wavelength (450 nm and 570 nm) in an ELISA reader (Biotek, USA). The concentration of RBD-specific antibodies (ng/mL) was quantified using isotype-specific anti-RBD monoclonal antibodies. Samples with an IgG antibody concentration ≥ 500 ng/mL (0.5 µg/mL) were considered to be seropositive, as reported previously [1].

### 2.3. Data Analyses

SARS-CoV-2 IgG-specific antibody responses in COVID-19-vaccinated participants were analyzed after each primary and booster dose. Mann–Whitney U tests were performed for statistical comparison. Plots and analyses were prepared using Graph Pad Prism (version 8.0).

## 3. Results

### 3.1. Vaccination

Among the primary vaccine recipients (n = 6300, including both dose 1 and dose 2), 2855 received ChAdOx1 nCoV-19, 121 received BNT162b2, 578 received mRNA-1273, and 2746 participants received a Vero-cell-inactivated vaccine (Table 1). A total of 59% (n = 3745) of all the primary vaccine recipients received booster doses either through homologous or heterologous approaches. Among those who initially received the ChAdOx1 nCoV-19 vaccine, 20.8% (n = 593) received a homologous ChAdOx1 ncoV-19 booster, while 47% (n = 1343) received a heterologous booster (third dose) with either mRNA-1273 or BNT162b2. Among the primary BNT162b2 recipients, 27.3% (n = 33) received the homologous BNT162b2 booster and 46.3% (n = 56) received the heterologous booster with mRNA-1273. Among the primary mRNA-1273 recipients, 27.9% (n = 161) received a homologous mRNA-1273 booster, while 21% (n = 122) received a heterologous booster; moreover, 19% received BNT162b2 and 2% received ChAdOx1 nCoV-19. Among those who received a Vero-cell-inactivated vaccine as their primary vaccine, only 0.4% received a homologous vaccine while 52% (n = 1435) received a heterologous booster, including ChAdOx1 nCoV-19 (14.3%), BNT162b2 (24.6%), and mRNA-1273 (13.0%). Overall, 5.5% (n = 347) of the 6300 vaccinees received a second booster (fourth dose); 343 of these received BNT162b2. Details of all primary and booster vaccines are shown in Table 2.

### 3.2. Antibody Responses After Homologous and Heterologous COVID-19 Vaccination Booster

**Figure 1:**
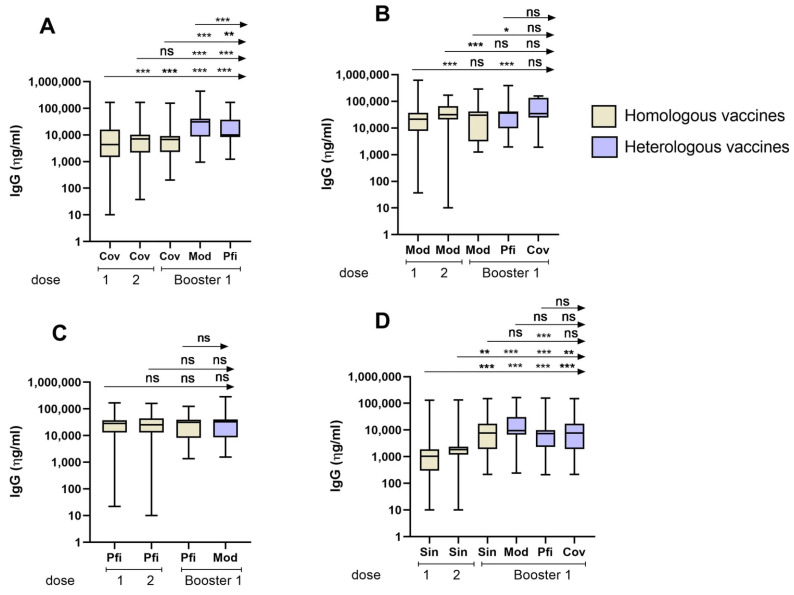
Antibody responses after homologous and heterologous COVID-19 booster vaccination. SARS-CoV-2 spike RBD-specific IgG (ng/mL) antibodies were measured in serum samples from individuals who received homologous or heterologous booster vaccines one year after receiving primary doses of ChAdOx1 nCoV-19 (n = 2711) (**A**), mRNA-1273 (n = 528) (**B**), BNT162b2 (n = 120) (**C**), or a Vero-cell-inactivated vaccine (n = 2746) (**D**). Blood specimens were collected and analyzed after 1 month (dose 1), 2/3 months (dose 2) after the first vaccination, and 1 month after the first booster vaccination. Box and whisker plots indicate median IgG concentration with minimum and maximum values at each time point. Statistical comparisons between homologous and heterologous booster responses were performed using the Mann–Whitney test (* *p* < 0.05, ** *p* < 0.001, *** *p* < 0.0001, ns = not significant; *p* > 0.05).

**Figure 2:**
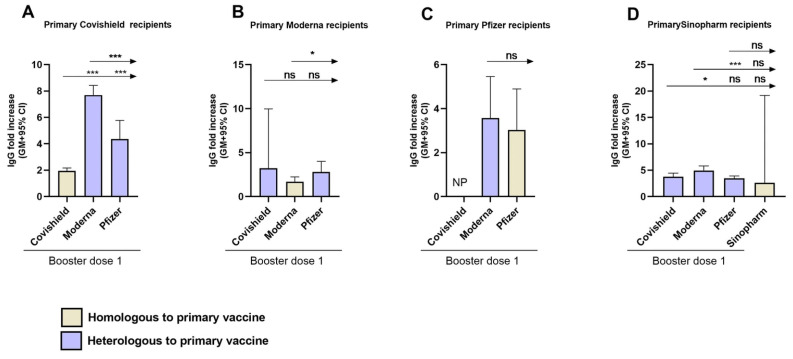
Magnitude of antibody responses after homologous and heterologous COVID-19 booster vaccination. SARS-CoV-2 spike RBD-specific IgG fold rise (geometric mean ± 95% confidence intervals) was measured after receiving the first booster doses in individuals who primarily received (**A**) ChAdOx1 nCoV-19 (booster, n: ChAdOx1 nCoV-19 = 592, mRNA-1273 = 1198, BNT162b2 = 142), (**B**) mRNA-1273 (booster, n: ChAdOx1 nCoV-19 = 11, mRNA-1273 = 160, BNT162b2 = 107), (**C**) BNT162b2 (booster, n: ChAdOx1 nCoV-19 = 0, mRNA-1273 = 56, BNT162b2 = 33), or (**D**) a Vero-cell-inactivated vaccine (booster, n: ChAdOx1 nCoV-19 = 352, mRNA-1273 = 352, BNT162b2 = 581, Vero-cell-inactivated vaccine = 7). Fold difference was calculated by dividing the antibody concentrations found after one month of booster vaccination by pre-booster. Booster responses are shown for homologous (beige) and heterologous (blue) vaccines. Statistical comparisons were performed using the Mann–Whitney test (* *p* < 0.05, *** *p* < 0.0001, ns = not significant; *p* > 0.05). NP = no participant.

To evaluate the humoral immune response following various primary and booster vaccinations, we measured IgG antibody responses across different vaccine groups (Figure 1). Overall, heterologous boosting was found to enhance IgG levels compared to homologous booster vaccines. Individuals who received two doses of ChAdOx1 nCoV-19 exhibited a significant rise in IgG levels (*p* < 0.0001) upon receiving a heterologous booster with either mRNA-1273 or BNT162b2 (Figure 1A). Both mRNA boosters induced comparable IgG responses, with no significant differences found between them. In contrast, compared to a second dose of ChAdOx1 nCoV-19, antibody responses did not increase further after a homologous ChAdOx1 nCoV-19 booster (*p* > 0.05). The BNT162b2 booster led to the highest IgG fold increase, followed by mRNA-1273, with both demonstrating a statistically significant difference compared to ChAdOx1 nCoV-19 (*p* < 0.0001, Figure 2A).

Participants who received a primary series of mRNA-1273 followed by a heterologous booster with BNT162b2 or ChAdOx1 nCoV-19 exhibited only a small increase in IgG levels. However, a significant rise in antibody responses was observed in BNT162b2-boosted individuals (Figure 1B). On the other hand, homologous mRNA-1273-boosted individuals maintained high IgG levels, indicating a sustained response with homologous boosting. The fold increase of IgG was comparable across all boosted groups, with no significant differences between heterologous and homologous boosters except for a slight increase observed with BNT162b2 (*p* < 0.05, Figure 2B).

Among individuals who received BNT162b2 initially, boosting with homologous (BNT162b2) or heterologous (mRNA-1273) vaccines did not enhance IgG levels further compared to dose 1 and dose 2 (Figure 1C). Statistical analysis showed no significant differences among these groups, suggesting that the choice of mRNA booster may not substantially impact IgG titers when the primary series is also mRNA-based.

A distinct trend was observed in individuals who received Vero-cell-inactivated vaccine as their primary doses (Figure 1D). After dose 1 and dose 2, a small rise in antibody levels was observed. However, after receiving both heterologous (BNT162b2 or mRNA-1273) and homologous boosters (Vero-cell-inactivated), IgG levels increased significantly (*p* < 0.0001). The strongest IgG magnitude (fold rise) of IgG was observed for mRNA-1273 boosting (*p* < 0.0001), followed by ChAdOx1 nCoV-19 boosting (*p* < 0.05, Figure 2D).

### 3.3. Longevity of IgG Response Following COVID-19 Vaccination Booster

**Figure 3:**
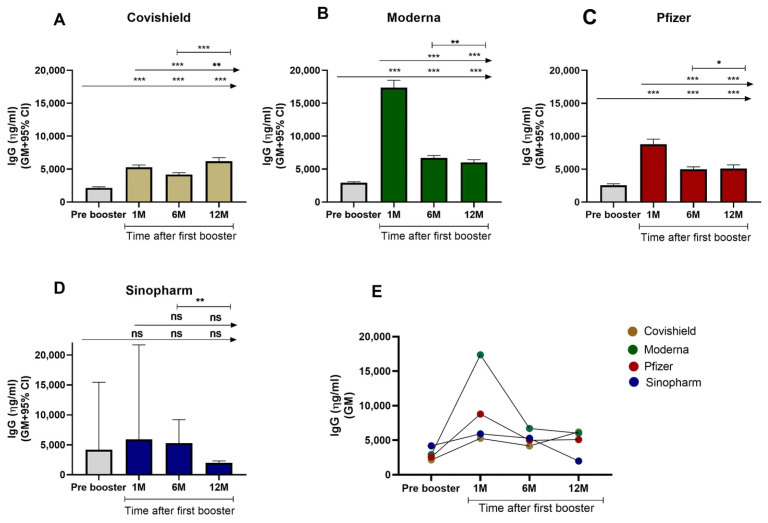
Longevity of antibody responses up to 1 year after four different COVID-19 booster vaccinations. SARS-CoV-2 spike RBD-specific IgG (ng/mL) antibodies were measured in participant serum samples at multiple time points: before booster vaccination (pre-booster), after 1 month (1 M), 6 months (6 M) and 12 months (12 M) post booster dose 1. The bar plots show geometric mean of IgG concentration with 95% confidence intervals for the participants received (**A**) ChAdOx1 nCoV-19 (n = 998), (**B**) mRNA-1273 (n = 1775), (**C**) BNT162b2 (n = 960) and (**D**) Vero-cell-inactivated vaccine (n = 10) as booster vaccine dose 1. The line graph (**E**) shows the kinetics of IgG antibody responses over time up to 12 months across different booster vaccine groups. Statistical analysis (**A**–**D**) was performed between different time points using the Mann–Whitney test (* *p* < 0.05, ** *p* < 0.001, *** *p* < 0.0001, ns = not significant; *p* > 0.05).

To evaluate the durability of the immune response after booster vaccination, we measured IgG levels at different time points (pre-booster, 1 month, 6 months, and 12 months post-booster) (Figure 3).

Among individuals who received ChAdOx1 nCoV-19 as their primary vaccine, IgG levels significantly increased at the 1-month post-booster timepoint (*p* < 0.0001) and remained elevated at 12 months (*p* < 0.01, Figure 3A). In those who received mRNA-1273 as their primary vaccine, booster administration resulted in a robust increase in IgG levels at 1 month post-boosting (*p* < 0.0001). Although a decline was observed at 6 and 12 months, IgG levels remained significantly elevated compared to pre-booster levels (*p* < 0.0001 at 12 months, Figure 3B). Similar to mRNA-1273 recipients, those with a primary BNT162b2 vaccination exhibited a substantial increase in IgG levels 1 month post-booster (*p* < 0.0001). However, there was a progressive decline at 6 and 12 months, though IgG levels remained significantly higher than pre-booster levels (Figure 3C). For individuals who received Vero-cell-inactivated vaccinations as their primary vaccine, IgG levels showed a modest increase at 1 month post-booster, but this increase was not statistically significant compared to pre-booster levels. The levels remained stable over time; however, after 12 months, IgG levels significantly declined compared to 6 months post-booster (Figure 3D).

A comparative analysis of IgG responses across all primary vaccine groups demonstrated that mRNA-1273 recipients had the highest peak IgG levels at 1 month post-booster, followed by BNT162b2 and ChAdOx1 nCoV-19, while Vero-cell-inactivated vaccine recipients showed the lowest response (Figure 3E). Over time, all groups exhibited a decline in IgG levels, with the most pronounced waning occurring in Vero-cell-inactivated vaccine and BNT162b2 booster recipients. Compared to other vaccine types, Vero-cell-inactivated recipients demonstrated a relatively flat IgG trajectory, indicating a limited booster effect.

### 3.4. IgG Response Following Second COVID-19 Vaccination Booster

**Figure 4:**
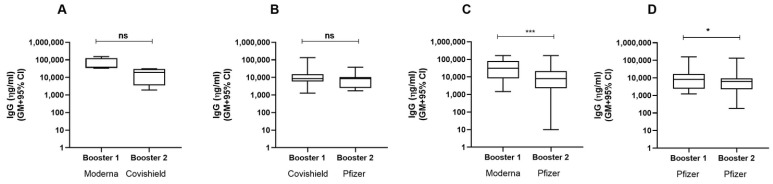
Antibody responses after administration of second booster vaccines one year after first booster vaccination. IgG concentration (ng/mL) specific for SARS-CoV-2 spike RBD was measured after second booster doses (ChAdOx1 nCoV-19 or BNT162b2) compared with their antibody levels following booster dose 1. The box plot represents the geometric mean with 95% confidence intervals of antibody concentration for participants who received (**A**) mRNA-1273 as booster 1 (n = 4) and ChAdOx1 nCoV-19 as booster 2 (n = 4), (**B**) ChAdOx1 nCoV-19 as booster 1 (n = 36) and BNT162b2 as booster 2 (n = 36), (**C**) mRNA-1273 as booster 1 (n = 154) and BNT162b2 as booster 2 (n = 153), and (**D**) BNT162b2 as booster 1 (n = 81) and BNT162b2 as booster 2 (n = 81). Statistical analysis was performed between one-month post-booster doses using the Wilcoxon test (* *p* < 0.05, *** *p* < 0.0001, ns = not significant; *p* > 0.05).

Only a small percentage of study participants (5.5%) received a fourth vaccine dose (a second booster); of these, 99% of them received BNT162b2. Thus, we grouped booster 2 recipients into 4 groups based on the type of first and second booster vaccines administered. In all cases, and surprisingly, IgG antibody responses decreased after the second booster doses compared to the first booster doses (Figure 4).

## 4. Discussion

This study provides a comprehensive evaluation of IgG antibody responses following primary and booster COVID-19 vaccination as well as immune durability over a 24-month period in a low-income country (Bangladesh). To the best of our knowledge, this is the first study to compare kinetics and magnitudes of antibody responses after four different booster vaccinations in Bangladesh. During the pandemic period in Bangladesh, the most widely used primary and booster COVID-19 vaccines were ChAdOx1 nCoV-19 (Covishield, Serum Institute of India), mRNA-1273 (Moderna), BNT162b2 (Pfizer-BioNTech), and Vero-cell-inactivated vaccines (Sinopharm). Our earlier studies demonstrated the decline of antibody levels six months following two doses of the primary vaccine [1] (Akhtar M et al., manuscript in submission). Decreases in vaccine-induced immunity coupled with the emergence of SARS-CoV-2 variants led to increases in breakthrough infections, prompting worldwide consideration for vaccine booster doses [26]. Due to shortages and unequal distribution of COVID-19 vaccines in developing countries, mixing or heterologous COVID-19 booster vaccinations were endorsed [14,27].

Our findings demonstrated key differences in booster-vaccine-induced immune responses across various primary vaccine groups. mRNA-based vaccines (Moderna and Pfizer-BioNTech) elicited the strongest and most durable IgG responses following a booster, while viral vector-based vaccines (ChAdOx1 nCoV-19) induced only moderate responses with noticeable waning over time. Individuals administered Vero-cell-inactivated vaccines exhibited the weakest response, highlighting the need for optimized booster strategies for this group. Overall, we have shown that heterologous boosting generally enhances IgG levels more effectively than homologous boosting, particularly for individuals who initially received viral vector or inactivated vaccines. Other studies have also shown that heterologous boosting strategies induce higher levels of anti-spike protein antibodies, increased neutralizing antibodies, and a more robust T-cell response compared to homologous vaccination schedules [13,27,28]. Among ChAdOx1 nCoV-19 recipients, boosting with either BNT162b2 or mRNA-1273 led to a significant rise in IgG levels compared to a homologous booster. This aligns with previous studies demonstrating the immunological advantage of mRNA booster vaccines in individuals primed with viral vector vaccines [28]. Similarly, among individuals who received the Vero-cell-inactivated vaccine or heterologous boosting, especially with mRNA vaccines, there was a marked increase in IgG levels, whereas a homologous booster provided minimal response. These findings underscore the limitations of inactivated vaccines in generating robust antibody responses and the need for mRNA-based heterologous boosting strategies in these individuals. Longitudinal analysis of IgG levels post-booster revealed that while all vaccine groups exhibited peak IgG levels at 1-month post-booster, there was a progressive decline over time. mRNA-based vaccines (BNT162b2 and mRNA-1273) induced the highest IgG responses at peak, with sustained antibody levels at 6 and 12 months.

Our study highlighted a significant decline in IgG antibody response after the fourth booster, particularly mRNA vaccine doses given one year after the third dose (booster 1). This is consistent with emerging evidence suggesting diminishing responses with repeated boosting, particularly in individuals who previously received mRNA vaccines. This decline aligns with studies reporting waning immune responses after a fourth dose [29,30]. One study in Israel also demonstrated a significant decline in IgG, IgA, and neutralizing antibodies in participants receiving the BNT162b2 vaccine [30]. In another study of elderly individuals who received a third dose of the COVID-19 vaccine, a decline in specific antibodies against the Delta and Omicron variants over five months was observed [31]. All of these data suggest that repeated COVID-19 vaccination might lead to waning immunity [29,31,32], prompting questions about optimal dosing schedules and long-term protection maintenance. We and other groups have reported the emergence of anti-inflammatory and tolerance-inducing IgG4 responses after administration of the third and fourth doses of mRNA [33,34]. In addition, we found that IgG1-to-IgG4 ratios decreased with increasing vaccine doses [33], which might be responsible for the lower immunity found after the administration of second booster doses.

Although this study provides valuable insights into booster-induced antibody responses, it has some limitations, including evaluating only IgG antibodies as a surrogate for immune protection. Further studies evaluating neutralizing antibody activity and T-cell responses are needed to fully understand vaccine-induced immunity. In summary, our findings highlight the critical role of heterologous mRNA boosters in enhancing IgG responses and can inform strategies to combat future pandemic. These data provide a strong foundation for optimizing booster vaccination policies to ensure sustained protection against COVID-19.

## Figures and Tables

**Table 1:** Demographic information of the study participants.

	Primary Vaccines (Dose 1 and Dose 2)			
	ChAdOx1 nCoV-19	BNT162b2	mRNA-1273	Vero-Cell-Inactivated
Number of participants, n	2855	121	578	2746
Age, median (IQR)	48 (55, 42)	44 (55, 35)	38 (47, 32)	35 (45, 23)
Sex, n (%)				
Male	1861 (65.19)	74 (61.16)	389 (67.31)	1810 (65.92)
Female	994 (34.82)	47 (38.85)	189 (32.7)	936 (34.09)
Participants received any booster vaccine, n (%)	1936 (67.82)	89 (73.56)	283 (48.97)	1437 (52.34)

**Table 2:** Primary and booster vaccination information of the study participants.

	Primary Vaccines (Dose 1 and Dose 2)			
	ChAdOx1 nCoV-19 n = 2855	BNT162b2 n = 121	mRNA-1273 n = 578	Vero-Cell-Inactivated n = 2746
Booster 1 (dose 3), n (%)				
ChAdOx1 nCoV-19	593 (20.8)	-	12 (2.1)	393 (14.3)
BNT162b2	142 (4.9)	33 (27.3)	110 (19.0)	675 (24.6)
mRNA-1273	1201 (42.1)	56 (46.3)	161 (27.9)	357 (13.0)
Vero-Cell-Inactivated	-	-	-	10 (0.3)
Booster 2 (dose 4), n (%)				
ChAdOx1 nCoV-19	4 (0.2)	-	-	-
BNT162b2	131 (4.6)	3 (2.5)	23 (3.9)	186 (6.8)
mRNA-1273	-	-	-	1 (0.04)
Vero-Cell-Inactivated	-	-	-	-

## Data Availability

The raw data supporting the conclusions of this article will be made available by the authors, without undue reservation.

## References

[1] Bhuiyan T.R., Akhtar M., Khaton F., Rahman S.I.A., Ferdous J., Alamgir A.S.M., Rahman M., Kawser Z., Hasan I., Calderwood S.B. (2022). Covishield vaccine induces robust immune responses in Bangladeshi adults. IJID Reg. Online.

[2] Stirrup O., Krutikov M., Tut G., Palmer T., Bone D., Bruton R., Fuller C., Azmi B., Lancaster T., Sylla P. (2022). Severe Acute Respiratory Syndrome Coronavirus 2 Anti-Spike Antibody Levels Following Second Dose of ChAdOx1 nCov-19 or BNT162b2 Vaccine in Residents of Long-term Care Facilities in England (VIVALDI). J. Infect. Dis..

[3] Doria-Rose N., Suthar M.S., Makowski M., O’Connell S., McDermott A.B., Flach B., Ledgerwood J.E., Mascola J.R., Graham B.S., Lin B.C. (2021). Antibody Persistence through 6 Months after the Second Dose of mRNA-1273 Vaccine for Covid-19. N. Engl. J. Med..

[4] Suthar M.S., Arunachalam P.S., Hu M., Reis N., Trisal M., Raeber O., Chinthrajah S., Davis-Gardner M.E., Manning K., Mudvari P. (2022). Durability of immune responses to the BNT162b2 mRNA vaccine. Med.

[5] Hoeve C.E., Huiberts A.J., de Gier B., Andeweg S.P., den Hartog G., de Melker H.E., Hahne S.J.M., van de Wijgert J., van den Hof S., Knol M.J. (2024). COVID-19 vaccination-induced antibody responses and waning by age and comorbidity status in a large population-based prospective cohort study. Vaccine.

[6] Habib M.T., Rahman S., Afrad M.H., Howlader A.M., Khan M.H., Khanam F., Alam A.N., Chowdhury E.K., Rahman Z., Rahman M. (2023). Natural selection shapes the evolution of SARS-CoV-2 Omicron in Bangladesh. Front. Genet..

[7] Nasif M.A.O., Sultana N., Rahmat R., Akther T., Nessa A., Jahan M. (2022). Co-circulation of Multiple SARS-CoV-2 Variants of Concern in Dhaka, Bangladesh, during the Second Wave of the COVID-19 Pandemic. Microbiol. Resour. Announc..

[8] Araf Y., Akter F., Tang Y.D., Fatemi R., Parvez M.S.A., Zheng C., Hossain M.G. (2022). Omicron variant of SARS-CoV-2: Genomics, transmissibility, and responses to current COVID-19 vaccines. J. Med. Virol..

[9] Roy D.N., Ali S., Sarker A.K., Islam E., Azam M.S. (2023). Acceptance of COVID-19 vaccine booster dose among the people of Bangladesh: A cross-sectional study. Heliyon.

[10] Liu Y., Zeng Q., Deng C., Li M., Li L., Liu D., Liu M., Ruan X., Mei J., Mo R. (2022). Robust induction of B cell and T cell responses by a third dose of inactivated SARS-CoV-2 vaccine. Cell Discov..

[11] Karaali R., Öykü Dinç H., İnanç Balkan İ., Can G., Keskin E., Çolak H., Daşdemir F.O., Aydoğan O., Budak B., Kaya S.Y. (2023). Homologous or heterologous COVID-19 vaccine schemes: Comparison of immune responses and side effects. Diagn. Microbiol. Infect. Dis..

[12] Fu J.Y.L., Pukhari M.H., Bador M.K., Sam I.C., Chan Y.F. (2023). Humoral and T Cell Immune Responses against SARS-CoV-2 after Primary and Homologous or Heterologous Booster Vaccinations and Breakthrough Infection: A Longitudinal Cohort Study in Malaysia. Viruses.

[13] Arunachalam P.S., Lai L., Samaha H., Feng Y., Hu M., Hui H.S., Wali B., Ellis M., Davis-Gardner M.E., Huerta C. (2023). Durability of immune responses to mRNA booster vaccination against COVID-19. J. Clin. Investig..

[14] Atmar R.L., Lyke K.E., Deming M.E., Jackson L.A., Branche A.R., El Sahly H.M., Rostad C.A., Martin J.M., Johnston C., Rupp R.E. (2022). Homologous and Heterologous Covid-19 Booster Vaccinations. N. Engl. J. Med..

[15] Barros-Martins J., Hammerschmidt S.I., Cossmann A., Odak I., Stankov M.V., Morillas Ramos G., Dopfer-Jablonka A., Heidemann A., Ritter C., Friedrichsen M. (2021). Immune responses against SARS-CoV-2 variants after heterologous and homologous ChAdOx1 nCoV-19/BNT162b2 vaccination. Nat. Med..

[16] Luvira V., Pitisuttithum P. (2024). Effect of homologous or heterologous vaccine booster over two initial doses of inactivated COVID-19 vaccine. Expert Rev. Vaccines.

[17] Robbiani D.F., Gaebler C., Muecksch F., Lorenzi J.C.C., Wang Z., Cho A., Agudelo M., Barnes C.O., Gazumyan A., Finkin S. (2020). Convergent antibody responses to SARS-CoV-2 in convalescent individuals. Nature.

[18] Hussain A., Hasan A., Nejadi Babadaei M.M., Bloukh S.H., Chowdhury M.E.H., Sharifi M., Haghighat S., Falahati M. (2020). Targeting SARS-CoV2 Spike Protein Receptor Binding Domain by Therapeutic Antibodies. Biomed. Pharmacother..

[19] Yang J., Wang W., Chen Z., Lu S., Yang F., Bi Z., Bao L., Mo F., Li X., Huang Y. (2020). A vaccine targeting the RBD of the S protein of SARS-CoV-2 induces protective immunity. Nature.

[20] Ciarambino T., Para O., Giordano M. (2021). Immune system and COVID-19 by sex differences and age. Womens Health.

[21] Bates T.A., McBride S.K., Leier H.C., Guzman G., Lyski Z.L., Schoen D., Winders B., Lee J.Y., Lee D.X., Messer W.B. (2022). Vaccination before or after SARS-CoV-2 infection leads to robust humoral response and antibodies that effectively neutralize variants. Sci. Immunol..

[22] Bredholt G., Sævik M., Søyland H., Ueland T., Zhou F., Pathirana R., Madsen A., Vahokoski J., Lartey S., Halvorsen B.E. (2024). Three doses of Sars-CoV-2 mRNA vaccine in older adults result in similar antibody responses but reduced cellular cytokine responses relative to younger adults. Vaccine X.

[23] Li Z., Liu S., Li F., Li Y., Li Y., Peng P., Li S., He L., Liu T. (2022). Efficacy, immunogenicity and safety of COVID-19 vaccines in older adults: A systematic review and meta-analysis. Front. Immunol..

[24] Desmecht S., Tashkeev A., El Moussaoui M., Marechal N., Perée H., Tokunaga Y., Fombellida-Lopez C., Polese B., Legrand C., Wéry M. (2022). Kinetics and Persistence of the Cellular and Humoral Immune Responses to BNT162b2 mRNA Vaccine in SARS-CoV-2-Naive and -Experienced Subjects: Impact of Booster Dose and Breakthrough Infections. Front. Immunol..

[25] Akhtar M., Basher S.R., Nizam N.N., Kamruzzaman M., Khaton F., Banna H.A., Kaisar M.H., Karmakar P.C., Hakim A., Akter A. (2022). Longevity of memory B cells and antibodies, as well as the polarization of effector memory helper T cells, are associated with disease severity in patients with COVID-19 in Bangladesh. Front. Immunol..

[26] Krause P.R., Fleming T.R., Peto R., Longini I.M., Figueroa J.P., Sterne J.A.C., Cravioto A., Rees H., Higgins J.P.T., Boutron I. (2021). Considerations in boosting COVID-19 vaccine immune responses. Lancet.

[27] Atıcı S., Soysal A., Gönüllü E., Aydemir G., Öner N., Alan S., Engin H., Yıldız M., Karaböcüoğlu M. (2023). Comparison of humoral immune response in heterologous and homologous COVID-19 booster vaccine groups using CoronaVac and mRNA-based BNT162b2 vaccines. Rev. Soc. Bras. Med. Trop..

[28] Lv J., Wu H., Xu J., Liu J. (2022). Immunogenicity and safety of heterologous versus homologous prime-boost schedules with an adenoviral vectored and mRNA COVID-19 vaccine: A systematic review. Infect. Dis. Poverty.

[29] Menni C., May A., Polidori L., Louca P., Wolf J., Capdevila J., Hu C., Ourselin S., Steves C.J., Valdes A.M. (2022). COVID-19 vaccine waning and effectiveness and side-effects of boosters: A prospective community study from the ZOE COVID Study. Lancet Infect. Dis..

[30] Canetti M., Barda N., Gilboa M., Indenbaum V., Mandelboim M., Gonen T., Asraf K., Weiss-Ottolenghi Y., Amit S., Doolman R. (2022). Immunogenicity and efficacy of fourth BNT162b2 and mRNA1273 COVID-19 vaccine doses; three months follow-up. Nat. Commun..

[31] Renia L., Goh Y.S., Rouers A., Le Bert N., Chia W.N., Chavatte J.M., Fong S.W., Chang Z.W., Zhuo N.Z., Tay M.Z. (2022). Lower vaccine-acquired immunity in the elderly population following two-dose BNT162b2 vaccination is alleviated by a third vaccine dose. Nat. Commun..

[32] Goh Y.S., Rouers A., Fong S.W., Zhuo N.Z., Hor P.X., Loh C.Y., Huang Y., Neo V.K., Kam I.K.J., Wang B. (2022). Waning of specific antibodies against Delta and Omicron variants five months after a third dose of BNT162b2 SARS-CoV-2 vaccine in elderly individuals. Front. Immunol..

[33] Akhtar M., Islam M.R., Khaton F., Soltana U.H., Jafrin S.A., Rahman S.I.A., Tauheed I., Ahmed T., Khan I.I., Akter A. (2023). Appearance of tolerance-induction and non-inflammatory SARS-CoV-2 spike-specific IgG4 antibodies after COVID-19 booster vaccinations. Front. Immunol..

[34] Irrgang P., Gerling J., Kocher K., Lapuente D., Steininger P., Habenicht K., Wytopil M., Beileke S., Schäfer S., Zhong J. (2023). Class switch toward noninflammatory, spike-specific IgG4 antibodies after repeated SARS-CoV-2 mRNA vaccination. Sci. Immunol..

